# Current anti-doping policy: a critical appraisal

**DOI:** 10.1186/1472-6939-8-2

**Published:** 2007-03-29

**Authors:** Bengt Kayser, Alexandre Mauron, Andy Miah

**Affiliations:** 1 Professor, Institute of movement sciences and sports medicine, Faculty of medicine, University of Geneva, Switzerland; 2 Professor, Institute of biomedical ethics, Faculty of medicine, University of Geneva, Switzerland; 3 Reader, University of Paisley, Scotland, UK

## Abstract

**Background:**

Current anti-doping in competitive sports is advocated for reasons of fair-play and concern for the athlete's health. With the inception of the World Anti Doping Agency (WADA), anti-doping effort has been considerably intensified. Resources invested in anti-doping are rising steeply and increasingly involve public funding. Most of the effort concerns elite athletes with much less impact on amateur sports and the general public.

**Discussion:**

We review this recent development of increasingly severe anti-doping control measures and find them based on questionable ethical grounds. The ethical foundation of the war on doping consists of largely unsubstantiated assumptions about fairness in sports and the concept of a "level playing field". Moreover, it relies on dubious claims about the protection of an athlete's health and the value of the essentialist view that sports achievements reflect natural capacities. In addition, costly antidoping efforts in elite competitive sports concern only a small fraction of the population. From a public health perspective this is problematic since the high prevalence of uncontrolled, medically unsupervised doping practiced in amateur sports and doping-like behaviour in the general population (substance use for performance enhancement outside sport) exposes greater numbers of people to potential harm. In addition, anti-doping has pushed doping and doping-like behaviour underground, thus fostering dangerous practices such as sharing needles for injection. Finally, we argue that the involvement of the medical profession in doping and anti-doping challenges the principles of non-maleficience and of privacy protection. As such, current anti-doping measures potentially introduce problems of greater impact than are solved, and place physicians working with athletes or in anti-doping settings in an ethically difficult position. In response, we argue on behalf of enhancement practices in sports within a framework of medical supervision.

**Summary:**

Current anti-doping strategy is aimed at eradication of doping in elite sports by means of all-out repression, buttressed by a war-like ideology similar to the public discourse sustaining international efforts against illicit drugs. Rather than striving for eradication of doping in sports, which appears to be an unattainable goal, a more pragmatic approach aimed at controlled use and harm reduction may be a viable alternative to cope with doping and doping-like behaviour.

## Background

Since the inception, in 1999, of the World Anti Doping Agency – Agence Mondiale Anti-Dopage (WADA-AMA) and its anti-doping regulation, athletes in several sports are obliged to keep the authorities informed of their day-to-day whereabouts so that they can be obliged to urinate in full view of another person for sample collection, without prior notice (see the website of WADA-AMA [1]). In accordance with the WADA-AMA "athlete whereabouts information guidelines", the websites of national anti-doping agencies now provide athletes with forms to fill out with daily details of where the athlete stays overnight and goes during the day (for example the USA anti-doping agency website [2]). Similar forms are being used in other countries. This practice seriously impinges on personal privacy and is unacceptable in any other setting except, perhaps, imprisonment. Yet it is considered ethically acceptable in elite sport, since it is meant to protect the noble principles of fair competition, which therefore trump the value of an individual's private sphere. Indeed, it is commonly argued that athletes must relinquish some personal privacy, in order for fair competition to be possible. Our inquiry draws on a developing body of literature within medical ethics that discusses sports related enhancement issues [3-6]. We raise questions about the degree of privacy violation that anti-doping organisations are entitled to request from athletes, on the basis of this sporting norm. We are doubtful about the rule that fair competition should trump fundamental liberties in the majority of cases and are concerned about the escalation of this requirement in contemporary elite sport. The implicit normative framework of elite sports is itself a complex ethical and ideological construct, whose analysis lies beyond the scope of this paper. However, we argue that this normative framework currently plays out into costly surveillance and medical testing practices that are increasingly at odds with the norms of medical ethics and with received notions of personal privacy.

Since the medical profession plays an important role in the war on doping, we need to analyse this situation in order to assess whether the physician's role in anti-doping is compatible with prevailing medical ethics. In this article we will argue that the moral and ethical foundations of the war on doping are doubtful at best. In response, we advance both theoretical and pragmatic arguments that oppose the current trend of intensified and increasingly costly efforts to limit the use of doping in sports. Specifically, we critically explore four main ethical justifications for anti-doping: 1) the level playing field argument, 2) protecting the athlete's health 3) the concern for professional integrity and 4) the concern about unnecessary risk taking. In response to these arguments and in view of fundamental inconsistencies within current anti-doping policy and its limited effectiveness, we propose a model of medically supervised doping which takes into account the moral responsibility of medical professionals.

## Discussion

### A Level Playing Field

The first foundation for anti-doping concerns the concept of fair-play. It is reasoned that athletes should compete on equal grounds [7,8]. One purpose of the rules of sports is to define the 'level playing field' on which athletes compete and thus to articulate the notion of fair-play. Currently, anti-doping policies are part of these rules since doping practices are typically seen as cheating. We do not question the need for rules in sports nor the possibility of finding workable 'level playing field' definitions. However, we do find the anchoring of today's anti-doping regulations in the notion of fair-play to be misguided.

Official thinking on these issues simply assumes the validity of the level playing field concept without coming to terms with the reality of widespread biological and environmental inequality. People differ in their biological capacities, which result from interplay between genome and environment. This also applies to athletes and their performance capabilities. Genetic predisposition is of prime importance in this respect even though the identification of these genetic traits is taking time [9]. In fact, even a simple genetic mutation may confer a performance advantage. For example, in one Finnish family, a mutation in the erythropoietin receptor has increased the sensitivity of erythroïd progenitor cells leading to high hematocrit. The clinical condition is mild and life span is unaffected. The family's most famous member, Eero Maentyranta, whose blood carries more haemoglobin and therefore more oxygen than that of the average male, won three gold medals in cross-country skiing at the 1964 Winter Olympics in Innsbruck [10]. This example reveals the importance of inherited characteristics for performance. Yet, it is treated very differently by conventional sports ethics policies when compared with for example pharmacological aids, even though neither example is 'earned' by the athlete. Apparently, prevailing sports ethics is unconcerned about this contradiction since 'natural' genetic variation is considered to be an acceptable (or irrelevant) inequality, whereas artificial enhancement is not. However, while WADA has recently signalled a concern about the use of genetic screening for performance [11], there are no strict prohibitions of such use. Nevertheless, it will be interesting to follow this development as the warning from the WADA comes just months after the commercialisation of the first genetic tests for performance, which are now being introduced to a range of countries [12].

In addition to genetics, several other contingent facts about the athlete's circumstances fail to be reflected adequately in the current ethical framework of anti-doping. For instance, depending on their nationality and sports speciality, athletes may differ enormously with regard to their access to care, supervision, and a high quality medical and technological environment [5,13]. Being a top athlete from a rich country is completely different from being an athlete from the developing world. There is certainly no evidence of equality of conditions here and there probably never will be. Furthermore, in a rich high-tech environment, an athlete may come as close as possible to doping, and sometimes into doping, all the while being medically supervised in a sophisticated technological environment.

These inequalities are further compounded by the possibility of undetected sophisticated doping. The recent cases surrounding the United States Bay Area Laboratory Co-Operative (BALCO) concerning the designer anabolic steroid, tetrahydrogestrinone (THG) [14], clearly show that, given sufficiently high stakes, inventive people will circumvent anti-doping strategies and may remain undiscovered, at least temporarily. It is relevant to note that the discovery of THG came as a result of an individual's 'good will' rather than the success of anti-doping laboratories. In 2003, a syringe filled with the substance was left anonymously at Dr Don Catlin's anti-doping lab at UCLA, from which his team was able to characterise THG and develop a test for it [15]. Presently, anti-doping relies predominantly on tests for substance groups that are available through prescription or that are known to the anti-doping laboratories as potential doping agents. Anti-doping cannot possibly develop tests for all substances that have ever been developed, especially those that never made it to full commercialisation and about which little is known. This confers force to the claim that anti-doping will always remain one step behind the dopers. Moreover, these circumstances give credence to the argument that doping tests are not effective if they lead merely to catching those athletes who do not have the best 'rogue' scientists working for them. The use by athletes from countries with less access to high-tech medical supervision during the 2004 Athens Olympic Games, of 'old' doping technology like the anabolic steroid stanozolol [16] suggests another dimension of this economic inequality. Since testing techniques for these older substances are well established, their users run greater risk of discovery than those who have access to newer more sophisticated molecules [5]. The response might be that the function of testing is as much a deterrent as a mechanism to ensure a level playing field. Indeed, one might claim that failure to detect all cheats is not an argument against striving to do so, since this would mean that perhaps all forms of regulatory systems are inadequate. However, we question this argument, for while it is common for anti-doping advocates to analogise their work to the criminal justice system, this analogy does not hold. In fact, sports are particular because their social value relies on whom is celebrated as the winners of competitions. In turn, it is presumed that these winners undertake their achievements by actions that merit praise or are virtuous. Such actions might include the discipline of training, the learning and acquisition of skills, or even a feel for the game that is somehow special. Yet, if the system is ineffective, then these crucial values are compromised. In contrast, normative systems designed to police society at large do not make high-minded assumptions about universal virtue and are, therefore, more resilient as regards the continued existence of transgressions. In addition, even though in elite sports repression may have led to a reduction in doping such is not the case in amateur sports and outside sports, where the available evidence clearly indicates continuous use of performance enhancing substances [17-22].

One more important problem concerns the potential of false positive tests. A recent report mentioned the potential of wrongly accusing an athlete of EPO use with the current testing procedures for EPO [23]. Anti-doping tests are just as much limited by sensitivity, specificity, precision and reliability as any other biomedical test and acceptable limits for certain products have to be set rather high to prevent false positives and therefore false negatives will continue to occur.

To summarize, we argue that the present concept of fair-play implicit in the war against doping fails to incorporate several other sources of inequality between athletes. Considering the continuous discovery of doping cases and the impossibility of eradicating doping practices, the basic inequality between undiscovered doped athletes and 'clean' athletes is likely to persist. These circumstances invite questions about what system of addressing the inequalities associated with performance enhancement would be most likely to optimise equality. While we do not consider that the discussion turns merely on an equality argument, the 'spirit of sport' criterion within the World Anti-Doping Code is used to give special value to fairness within sport. It is used as an argument on which anti-doping is justified: to ensure athletes are playing the same game. We suggest that, from the perspective of equality, supervised doping practice is likely to provide the greater prospect of ensuring equality of competition. On such a system, competition results would be based on some system of merit, rather than the undeserved inequalities arising from, say, genetic capacities. Critics might argue that scientists, rather than athletes, earn such advantage and that this kind of achievement is not relevant to sports. However, elite athletes are also constituted by scientific knowledge and this is a valued aspect of contemporary sport. As such, translating doping enhancements into earned advantages – having the best scientists on one's team – would more closely align to the values of competition than leaving it all to chance, unequal access to illicit practice, and the cleverness of undetected cheating.

### Protecting the Athlete's Health

The second ethical foundation for anti-doping is the protection of the athlete's health. It is reasoned that anti-doping control is necessary to prevent damage from doping. Even though we endorse the principle of concern about the health of the athlete, there are reasons to question the particular form of this principle as related to anti-doping policy.

### The Concern for Professional Integrity

When advocating the need for anti-doping in sport, a strong claim seems to emerge from the values implied by the medical professional's role and the proper role of medicine. There are two parts to this claim; the first relates to a stance on the legality of medical standards, which rejects doping methods because they are instances of medical intervention for non-therapeutic purposes. According to a commonly held position today, medical practice should be either preventive or therapeutic, i.e. aimed at preventing or treating disease, but should not use bio-medical technology for human enhancement. Indeed, much discussion in contemporary bioethics seems particularly concerned about the legitimacy of this conceptual distinction, though it is reasonable at least to indicate that such distinctions are made within medical practice, either because of the need to ration treatment or because health care providers do not consider enhancement to correspond with the proper role of medicine. Of further concern is that a particular doping practice has not been approved for use with healthy persons (such as athletes) and so has not benefited from the extensive clinical trials normally necessary before a therapeutic substance can be used. This is why, according to current anti doping policy, doping might be used legitimately with a therapeutic objective to increase the rate of repair of injury, but not if there is no medical need as such. In this sense, the use of doping methods to enhance performance is not sensible to many medical professionals because little is known about their effects on people who do not suffer from the very specific condition(s) for which the intervention was designed and tested. However, this view is not reflected in the wide spread use of off-label prescriptions. While the risks associated with such practice might be acceptable in a therapeutic context [24], it is deemed unacceptable in the realm of enhancement for sport. This is a salient point, since an advocate of doping cannot simply map onto sport substances that are already in existence for therapeutic use. Thus, we cannot claim that a specific form of, say, an anabolic steroid be granted permission for use by athletes. Rather, our claim would require approval for the development of an anabolic substance or dosage scheme designed and adapted specifically for athletes. The implications of this claim are quite different from advocating an uncritical acceptance of substances that already exist.

However, the ethical force of this point arises in the second part of the claim, which relates to the principle of non-maleficience, a principle that applies to all health professionals. In view of this principle, the ethics of anti-doping justifies itself on the basis that the counter-position would *require *medical professionals to use medical products in a way that might lead to greater harms for the patient or because it might compromise the physician's personal integrity. Thus, one might suggest that such risks are different from those an athlete takes when choosing to, say, go horse riding, since the latter does not require prior medical intervention before taking part. At most, it might involve some form of approval that the participant is in good health. In contrast, under medically supervised doping, a physician is making possible the enhancement by intervention and so undertakes a duty of care when treating the athlete. The difficulty with this claim is that sports physicians *already *engage in such practices when repairing athletes. Consequently, to reject 'enhancement' on this basis fails to take into account the bio-cultural character of health: that making people well always involves making them well for something that involves a whole range of risks. While it might be unreasonable to claim that all physicians have an obligation to enhance athletes, one would nevertheless recognise as legitimate a physician's choice to facilitate such a lifestyle. Indeed, the remaining arguments that might counter this view would involve some concern about the scarcity of resources, though sports are unlikely to rely on public funding for this purpose.

### The Concern about Unnecessary or Irrelevant Risks

The second concern about protecting the athlete's health that is often used to justify anti-doping is that doping risks are qualitatively different from other sporting risks, because the former are unnecessary and irrelevant. This view takes into account the fact that elite sports are not innocuous [25,26]; participation may lead to serious health problems. Consequently, such practices are not considered unambiguously health promoting. For example, soccer comes with high risks for knee and ankle problems, well beyond that of the general population, especially in elite players [27]. Boxing, in its present form, is well known to be dangerous for the CNS [28]. In ice hockey and American football spine injury is frequent [29]. These risks – unlike doping risks – are often characterised (and justified) as a necessary part of the competition. However, the various sports are not defined by their essential nature; rules can be changed to make them safer. For example, boxing has made a number of rule changes over the years to reduce the potential for serious injury. But there is often a limit to reducing risk in this way, since excessive risk reduction could undermine the value typically attached to a particular sport. For instance, if one seeks to free climb a particular mountain route, then the practice is possible only by accepting the rule that no safety support is used. If this rule is not maintained, then the claim that one has climbed freely cannot be made. Thus, if the rules are changed, then the type of experience changes along with the values associated with it. While medical professionals will strive to make sports as safe as possible, there are certain risks that must be accepted in order to have the games take place.

For many practices, the claim about logical necessity and relevance cannot be advanced in relation to doping: one can undertake free climbing without using some form of doping. An interesting case arises when considering extreme performances. For instance, there are some forms of performance that are not possible without some form of technological enhancement. Perhaps for some climbers supplemental oxygen for climbing Mount Everest falls into this category. In these kinds of activities, enhancement has a contested status, though might be seen as a constitutive element of the performance in the same way that a tennis racquet is a constitutive technology of playing tennis. However, doping practice might make possible the experience of certain physical achievements that are simply not possible without the technology. Indeed, one might suggest that the level of competition in many sports is so high that being competitive requires a wide range of sophisticated technological assistance to be used in training. Therefore the notion that current elite sports competitions only test some naturally inherent ability of athletes does not reflect reality.

However, the more salient point is that the level of risk one accepts within the practices we enjoy cannot be prescribed by the moral norms of the medical profession. The kinds of risks one takes in daily life are determined through a complex, personal value system that can often appear inexplicable – such as the motivation for jumping out of aeroplanes or deep sea diving. It is problematic to make such value systems accountable to the moral judgement of the medical profession. Indeed, one conception of a health care system (which we advocate here) would suggest that one of its functions is precisely to care for the risks people freely take in their daily lives.

The key question is whether any rule or enhancement is 'sufficiently safe', rather than absolutely safe. We believe that doping *cannot *be sufficiently safe as long as it is prohibited and that this fact has a direct bearing on the integrity of medicine and the physician's commitment to maintain this integrity. Yet, under appropriate supervision, this risk could be more easily justified. Thus, a physician cannot simply assume that doping is, per se, more dangerous than the risks of engaging in elite sports. The risks of every doping technology must be assessed. In turn, this is especially difficult for an illegal practice whose risks are not well described, since they are largely hidden. For instance, the risk of well-controlled use of erythropoietin in elite sports is not well known, since only anecdotal information is available [30]. The use of dexamphetamine is likely to be dangerous, but scientifically sound data are scarce [30]. More data exist on anabolic steroids [30,31], but again secrecy prevents an evidence based assessment. Furthermore, in a context of prohibition and penalties for use that discourage scientific assessment of the risks, declaring that doping is dangerous becomes, to some extent, a self-fulfilling prophecy, since doping often happens without proper medical supervision or evidence from sound clinical trials. In elite sports there may at least be some medical supervision, possibly of good quality. This is not the case for the general population, which may result in serious health problems for a much greater number of subjects. Indeed, recent reports on the use of illicit pharmacological means to enhance performance in amateur sports are alarming with regard to the high prevalence of these practices [19,20,30,32-35].

### Response to the Protection of Health Arguments for Anti-doping

We propose that allowing medically supervised doping within the framework of classical medical ethical standards, particularly with regard to the principle of non-maleficience, would potentially have a number of positive consequences.

Firstly, it might lead to a clearer view of what is dangerous and what is not. At present doping is largely hidden and its epidemiology unknown. Additionally, the war on doping may have adverse effects of its own. Doping control leads to shifts in behaviour that entail an increased health risk. The detection of oil-based esters of nandrolone, belonging to a class of anabolic steroids with little side effects and low risk for hepatic disease has led to the use of oral analogues with more side effects, but more rapidly eliminated from the body and thus less easy to detect [36]. Now that recombinant erythropoietin is detectable, there is a shift to the use of other oxygen carrying capacity enhancing drugs, with higher potential health risks [37]. These consequences of anti-doping practices may thus paradoxically introduce more health problems than they prevent.

Secondly, elite sports activity often results in health problems that need specific attention. Sometimes, managing these health problems involves pharmacological interventions that are normally considered doping. The boundary between therapeutic and ergogenic (i.e. performance improving) use of pharmacological means is quite blurred and poses important problems to the controlling bodies of anti-doping practice and athletes' sports physicians [38]. Several substances can be used for medical reasons but are proscribed when the athlete is healthy or in competition. These rules for *therapeutic use exemption *(TUE) lead to complicated and costly administrative and medical follow-up [39]. They may even lead to athletes being denied medical care corresponding to a best practice standard. Cyclists with documented asthma could not be treated optimally because of the strictness of the rules [40]. Medically supervised doping would erase this dual identity of molecules – legitimate therapeutic agents vs. illicit doping – and thus eliminate these additional burdens. This would have to be put into the broader context of non-therapeutic use of substances or practices for reasons of human enhancement in general. Although such practices generate much uneasiness today, they need to be addressed frankly as the diversity and scope of human enhancement is bound to increase.

An example of accepted athlete's enhancement is a surgical procedure originally invented to repair injury of the ulnar collateral ligament of the elbow in baseball pitchers. Anecdotal evidence suggests that this procedure often allows pitchers to perform even better than before they were injured. In this case, the repair of athletes – along with the process of recovery through exercise – works to provide a 'better than well' performance outcome, without giving rise to any moral concern about unfair advantages. While this procedure now has a considerably greater success rate, its development in the 1970s was considerably more experimental and hence dangerous [41].

Thirdly, the concern about doping is largely disingenuous, if it is supposed to reflect a genuine moral concern for health. There is no lack of moral entrepreneurs, poised to preach the war on doping: sports authorities, politicians, opinion leaders, ethicists, and the media. They claim the moral high ground by waging what has become, in effect, what social scientists call a "symbolic crusade" [42]. Yet, while high-level sports is touted as embodying the positive values of health, meritorious effort, harmonious development of body and mind, this downplays the very real health risks of elite sports as well as accepted levels of foul play with considerable health damage in certain sports such as soccer or ice-hockey. Today's medical reality of high-level athletics little resembles the quaint image of an ideal harmony between beauty, strength and health dreamed up by the early Olympic movement. Elite sports have become thoroughly alien to the sort of physical exercise that is a legitimate general public health concern. In addition, high-level athletes are singled out for attention and their health-related behaviours subjected to an invasive scrutiny that would be impractical – and unethical – if it were applied to the general public.

The war on doping diverts scarce resources towards a program of intense and intrusive health surveillance for the few, which makes no sense in terms of public health, if only because the fraction of the population that engages in elite sport is very small. The problem is all the more obvious when compared to the frequent doping practices in amateur sport [18,43]. Indeed, the recent statement on performance-enhancing drug use by the American Academy of Pediatrics [44] emphasises the broader public-health rationale that should govern anti-doping strategies. It argues that the use of such substances is far broader than elite sport and focusing specifically on this area neglects the many other ways in which substances are used in ways that are dangerous. Doping is not just a sports issue, and therefore does not justify a sports-only approach [45]. In this era of anti-doping, a black market in substances such as anabolic steroids has developed, often of dubious quality. Dangerous practices have emerged, such as sharing syringes, leading to risk of HIV or hepatitis virus infection [17,21,22,46,47]. We should be concerned about the health of this much larger fraction of the population, instead of investing so much effort and money in surveillance of small numbers of often medically well supervised elite athletes. On this view, a drug testing programme is not the most effective way to curtail the use of performance enhancing (or lifestyle improving) substances. Rather, resources should be invested into understanding the shift in cultural values associated with biological modification and the culture in which doping practices emerge. Merely testing athletes attends only to the consequences of such a culture.

### The Cost of Anti-doping : Who Pays ?

We acknowledge the need for rules in sports. The principle of the adherence to a set of rules, including the prohibition of doping is, in itself, not problematic when considering the practice of sports. Houlihan [48] for example articulates the 'keep the rules' argument as part of an agreement that has social weight. However, one problem arises when the application of these rules is beset with diminishing returns: escalating costs and questionable effectiveness. As argued above we believe that the ethical foundation of the prohibition of the use of ergogenic substances in sports is weak at best. Therefore, we find that the increasing cost for the practice of anti-doping raises an ethical dilemma of greater importance and relevance than the ethical arguments advanced as the foundation for anti-doping practices.

Elite athletes only represent a small fraction of the global population but the resources of anti-doping almost exclusively go into testing of these athletes. The WADA-AMA budget amounted to 21 million dollars in 2004 [1]. According to its statutes, as of January 1st 2002, WADA-AMA's funding is sourced equally from the Olympic Movement and the governments of the world at least until 2007 [1]. The budget of the Swiss Anti-Doping Commission for 2004 was about SFr. 1.5 million whereof SFr. 800,000 came from the government [49]. The budget of the USA anti-doping agency in 2003 was 10 million dollars [2]. The UCI (Union Cycliste Internationale) spent 1.4 million Swiss francs directly on doping controls and testing in 2003 [50]. The overall world wide cost of anti-doping is difficult to estimate but is likely to be high in the light of the number of athletes concerned. It will probably increase further as the complexity of the analysis increases and the coverage of the world's elite athlete population improves. Today, the rich countries can pay the bill for the increasingly costly practice of doping control, but the developing countries cannot. There is money coming through international federations like the IOC, but increasingly, resources will accrue from governmental sources. Even though today the contribution asked from developing countries is small, especially in those countries the priorities should lie elsewhere from a public health perspective. Furthermore, we have seen that in the competition between increasingly sophisticated doping and anti-doping technology, there will never be a clear winner. Consequently, such a futile but expensive strategy is difficult to defend, especially since the much larger fraction of the population that engages in behaviour like use of anabolic steroids and needle sharing is a real health issue [21] and does not get the resources necessary for prevention and harm reduction.

Doping shares several characteristics with general substance abuse. Even in a repressive environment substance abuse persists, with potential harm because of the need to hide the abuse. The highest sanction for an athlete, whose doping practice is discovered, is a lifetime exclusion from competition, which is not enough to scare all athletes away from doping. The political and economic incentive, along with the personal quest for money, fame or the thrill of winning is so high that risk taking is likely to continue. As long as the rewards of competition remain high and the consequences of being caught are merely exclusion from competition, the likelihood of athletes using doping will remain high [5]. In addition, truly deterrent penalties would have to be as severe as sanctions for major crimes, which is indefensible in terms of social ethics.

## Special Cases

### Doping Control on Cannabinoid Use

There are additional inconsistencies in the foundation of the world-wide war on doping. If anti-doping were purely addressing the unfair advantage of an ergogenic intervention, anti-doping should be focussing on the control of the use of ergogenic substances only. Cannabis (marihuana, hashish) and its active substance THC are not performance enhancing; THC is probably merely deleterious for performance for any elite sport activity [51]. At present the WADA-AMA rules do not allow for traces of THC metabolites in urine, even though it is well known that these metabolites are found in urine well after the psychophysiological effects of the substance have subsided. Why then test for the substance? We believe that the inclusion of control on THC goes beyond the declared goal of anti-doping. Moreover, justification of their inclusion on the claim that athletes are role models is problematic, as this places an unreasonable burden on athletes, compared with other public figures like musicians, politicians or actors whom are not required to undergo such tests. Our point is that the intrusive monitoring of athletes actually undermines their status as role model, since it stigmatizes athletes as people who, without surveillance, will behave improperly. Thus, the burden is unreasonable not because it is unfair, but because it constitutes an attempt to orchestrate role model status which we consider to be deceptive and antithetical to what role models should be. In any case, there is no obvious reason for why testing for THC or similar drugs should be a matter of public concern, unless one also requests tests from other such public figures. If the response is that testing should be applied to other such people, then at least part of our claim would be redundant. However, we believe that there are good reasons for why such surveillance practices would be quite inappropriate in a liberal society. One might also raise questions about the role model status of most athletes. After all, while all competitive athletes are subject to anti-doping rules, only a few have a high public profile or a high salary. The majority have no greater public role than, say, a teacher or a parent. Yet, we do not hear pleas to test these and other people for illicit substances on account of their being role models.

### Accepted technology

The use of recombinant erythropoietin for enhancing the oxygen carrying capacity of the blood is prohibited in competitive sports. The alleged reason is clear, since it is accompanied by higher oxygen uptakes and improved endurance performance. Altitude exposure has a similar effect and leads to a natural increase in hematocrit and an increase in oxygen carrying capacity. Nowadays, altitude training camps, often touted with the slogan "sleeping high and training low" are popular [52]. Modern technology allows for the simulation of altitude with the use of hypobaric and normobaric hypoxia. Costly adaptations of sleeping quarters allow sleeping at virtual altitudes and several federations now have these facilities. Even individual athletes who can afford it have altitude sleeping rooms at home [52]. Since it is the body itself that brings about the increase in hematocrit when exposed to hypoxia, athletes are for now allowed to use this technology even though its objective is to gain "undeserved" advantage, just as with erythropoietin doping, and there are no long term data on its alleged innocuousness. Again, this is a challenge to equity, since many athletes cannot afford nor have access to such technology. Probably in part in response to this, WADA-AMA recently considered whether such technology should be banned, though has concluded that it should not. This outcome reinforces the inadequacy of anti-doping measures, since the difference between using these techniques and happening to live in a high-altitude locality is ethically irrelevant.

Other permitted technologies reflect a similar hypocrisy in anti-doping rules. For example, electrical muscle stimulation is increasingly used, either in preparation before a competitive event, or after. German athlete Wojtek Czyz won three gold medals (100 and 200 metres, and the long jump) at the 2006 paralympics in Athens after having trained with a unique, commercially unavailable electrical muscle stimulator developed for international space station use [53]. Many sports involve high tech material from swim suits and running shoes to futuristic bicycles. There is certainly no equitable access to these technologies for rich and poor alike [54]. The usual response to this comparison is that these forms of performance enhancement provoke a physiological response while doping methods a pharmacological one. Yet, this is not the justification for distinctions made within anti-doping policy. Indeed, we suggest that it reveals a dubious essentialism about what it means to be human that relies on claims about what is 'normal' or 'natural' for people to exhibit physiologically. We argue that sports have never been a test of merely 'natural' capabilities, but that they have always been constitutively technological, whether this involves specific artefacts or simply the application of scientific knowledge. This interaction between potentiality and environment is consistent with critical views of human genetics. Moreover, the difficulty with a commitment to essentialist views about natural capacities is made more apparent through the application of genetic technologies to sport specifically.

### Genetic Technology

Thus, one further challenge that lies ahead for the world of doping exacerbates the need for reform in anti-doping ethics: gene transfer. Also known as 'gene doping', this new form of potential performance enhancement has received considerable attention in recent years. While there is much scientific dispute about the science and current feasibility of gene doping [55,56], its prospect does alert us to the inadequacy of current strategies on doping control. Since some gene doping techniques might be undetectable in urine or blood in principle, one may wonder whether current approaches to doping are at all practical in an era where there can be no realistic expectation of catching all cheats. This might also move the war on doping to a stage of technical sophistication that might make it financially difficult to sustain. In addition, the broader social interest at stake with respect to gene transfer technology would give a new perspective to the question of what kinds of performances are legitimate in sport and how this ties in to concepts of equity, fair-play and deserved merit.

### What are the Risks of Leaving Doping Choices to the Athlete?

Even though it is presently unrealistic to abolish anti-doping in sport, let us briefly discuss what the hypothetical consequences would be if the use of doping were allowed. Would there be an important increase in death rate among athletes? Would there be many (more) athletes willing to take deadly risks? Would there be more chronic illness and shorter life span after cessation of an athletic career? If doping were allowed under the conditions we discuss, including an ethical framework based on the principle of non-maleficience, we would probably see an increase in the use of ergogenic drugs, but this need not to lead to an increase in morbidity and mortality. The example of the widespread use of doping in the former East-German republic [57,58] reflects the secret and coercive nature of state mandated doping, a framework widely different from the one we propose. Our proposal for monitored performance enhancement would ensure that athletes are better informed about the risks they take and transparency of these practices would limit the possibilities for a given nation from taking advantage of their athletes. Furthermore, taking doping out of hiding may have positive effects beyond the restricted world of elite sports. Indeed, the practices in the amateur sports world might become less hazardous and thus overall incidence of health problems from doping use might actually decrease. Unfortunately, it seems impossible to test this hypothesis in the current political climate, since there is hardly any interest in re-evaluating the ethical foundation of doping. Moreover, as Houlihan demonstrates [48], there has been no sustained open discussion of the ethical foundations of anti-doping since it began in the 1960s. If one were to compare this with other policy debates in science, medicine and technology, the situation is radically inadequate.

### What should the Physician's Role in Elite Sports be?

Suggestions about anti-doping reform have specific implications for medical professionals working with athletes. Yet, even within the present framework of anti-doping, problems arise that invite critical scrutiny of the established model. The current ethical framework of competitive sports is not without problems for the sports physician. As early as 1983, Thomas H. Murray, president of The Hastings Center (a leading institute for ethics), former United States Olympic Committee adviser, and present Chair of WADA-AMA's Ethical Issues Review Panel, argued that the conditions surrounding the physician's involvement with elite sport place undue pressure on their decision making capacities [59]. Often, the coach's or sponsor's interests take precedence over the physician's professional judgement about what is best for the athlete. On this basis, Murray argues that standards of best practice are often unclear or non-existent.

We believe that, in agreement with prevailing ethics of the medical profession, the role of the physician involved in the athlete's health supervision should be one of preserving the athlete's autonomy, which entails a balance between ensuring that treatment leads to the highest degree of present and future health, while acknowledging the athlete's interest to maintain a chosen style of life. Inevitably, there will be situations in which performance optimisation will conflict with the preservation of health just as it is already present today when therapeutic measures are applied to keep an athlete in the game despite an existing injury. Ethical reasoning should be based on proportionality, assessing the benefits and risks as objectively as possible. Admittedly, this is not an easy task, since it requires a process of negotiation to face the difficult question about what kinds of health risks are acceptable for an athlete to take. While further elaboration on this is beyond the scope of this paper, we would suggest that the solution lies partly in the structures of sport that permit such risk taking. Nevertheless, we believe that by carefully helping an athlete enhance her performance (by utilising currently banned methods), in keeping with the principle of autonomy, using any safe technology available, the physician should again become the direct partner of the athlete in pursuit of ever increasing performance. As a result, a physician in the role of caring performance enhancer should be accountable for ill effects from the use of any medical technology. This would be analogous to the usual role of physicians. They are free in their choice of intervention, pharmacologic or other, as long as these are in agreement with current medical knowledge and without disproportionate iatrogenic ill effects. Rather than speculate on anti-doping test procedures, resources should be invested into protecting the integrity of physicians who make such judgements. Without clear regulation, it is possible that coaches could appoint 'performance inclined' physicians to ensure maximum competitiveness in their athletes. Waddington [60] recognises that much more thought is needed to establish principles of good practice concerning the role of sports physicians. Perhaps independent physicians whose status is comparable to other sports officials, is the most suitable strategy through which to develop this more ethically rigorous requirement.

## Conclusion

Clearly, further questions need addressing to more fully explore our criticisms of current anti-doping and our proposal for alternatives. For instance, one might ask which athletes would qualify for doctor-assisted doping, whether there would be age limits or limits to performance levels. Moreover, it is necessary to explore matters of control and regulation and whether an organisation like the WADA-AMA remains the most suitable model. Sports are increasingly important for economic and political reasons. To a sizeable extent, elite sport is a self-sustaining enterprise, with significant financial returns from advertisement, media and audience revenues. As such, it could be argued that the war on doping is an internal matter of the sports community, provided that it foots the whole bill for anti-doping control. But in fact considerable public funds go into sports too, for fundamentally sound reasons such as health promotion. The increasingly expensive doping control is also paid by governmental sources, and not only by the sports enterprise itself. Moreover, the ethical foundations of sport are also a matter for public debate and, like for other ethical policies in society, there should be mechanisms ensuring accountability of policy to the broader public. For each of these reasons, the war on doping becomes a public issue as well. Hence, its consequences have to be seen from a public health perspective. We believe that current anti-doping does not adequately prevent damage from doping in sports, that it creates health problems of its own, and diverts health-care resources from more worthwhile pursuits. In addition, the role of the physician in sports and in doping control poses serious ethical dilemmas. We believe that allowing medically supervised doping rather than absolute bans would provide a sounder foundation for sports physicians to exercise their responsibility and maintain their health care obligations.

## Competing interests

The author(s) declare that they have no competing interests.

## Authors' contributions

BK, AMn and AM contributed equally to the paper. All authors have seen and approved this version of the manuscript.

## Pre-publication history

The pre-publication history for this paper can be accessed here:


